# Study Protocol for a Global Survey: Awareness and Preparedness of Hospital Staff Against Coronavirus Disease (COVID-19) Outbreak

**DOI:** 10.3389/fpubh.2021.580427

**Published:** 2021-07-01

**Authors:** Ahmad Taysir Atieh Qarawi, Sze Jia Ng, Abdelrahman Gad, Mai Ngoc Luu, Tareq Mohammed Ali AL-Ahdal, Akash Sharma, Vuong Thanh Huan, Nguyen Lam Vuong, Gehad Mohamed Tawfik, Mohammad Rashidul Hashan, Shyam Prakash Dumre, Sherief Ghozy, Hosam Waleed Shaikhkhalil, Mona Hanafy Mahmoud, Shamael Thabit Mohammed Alhady, Nguyen Hai Nam, Sheikh Mohammed Shariful Islam, Chris Smith, Peter Lee, R. Matthew Chico, Sharon Cox, Kenji Hirayama, Nguyen Tien Huy

**Affiliations:** ^1^Lower Westchester Medical Associates, P.C., Mount Vernon, New York, NY, United States; ^2^Online Research Club (http://www.onlineresearchclub.org), Nagasaki, Japan; ^3^School of Medical Sciences, University Sains Malaysia, Kelantan, Malaysia; ^4^Faculty of Medicine, Ain Shams University, Cairo, Egypt; ^5^Department of Internal Medicine, University of Medicine and Pharmacy at Ho Chi Minh City, Ho Chi Minh City, Vietnam; ^6^Department of Public Health, Faculty of Medicine, Jordan University of Science and Technology, Irbid, Jordan; ^7^University College of Medical Sciences & Guru Teg Bahadur Hospital, Dilshad Garden, India; ^8^Faculty of Medicine, Pham Ngoc Thach University of Medicine, Ho Chi Minh City, Vietnam; ^9^Faculty of Public Health, University of Medicine and Pharmacy at Ho Chi Minh City, Ho Chi Minh City, Vietnam; ^10^Government of Bangladesh, Ministry of Health and Family Welfare, Dhaka, Bangladesh; ^11^Central Department of Microbiology, Tribhuvan University, Kathmandu, Nepal; ^12^Faculty of Medicine, Mansoura University, Mansoura, Egypt; ^13^Faculty of Medicine, Islamic University of Gaza, Gaza, Palestine; ^14^Faculty of Medicine, University of Gezira, Wad Medani, Sudan; ^15^Division of Hepato-Biliary-Pancreatic Surgery and Transplantation, Department of Surgery, Graduate School of Medicine, Kyoto University, Kyoto, Japan; ^16^Institute for Physical Activity and Nutrition, Deakin University, Melbourne, VIC, Australia; ^17^Institute of Tropical Medicine and School of Tropical Diseases and Global Health, Nagasaki University, Nagasaki, Japan; ^18^P.N. Lee Statistics and Computing Ltd, Surrey, United Kingdom; ^19^Department of Disease Control, Faculty of Infectious and Tropical Diseases, London School of Hygiene and Tropical Medicine, London, United Kingdom

**Keywords:** awareness, preparedness, COVID-19, hospital staff, global survey

## Abstract

**Background:** The outbreak of Coronavirus disease (COVID-19) caused by a novel coronavirus (named SARS-CoV-2) has gained attention globally and has been recognized as a Public Health Emergency of International Concern (PHEIC) by the World Health Organization (WHO) due to the rapidly increasing number of deaths and confirmed cases. Health care workers (HCWs) are vulnerable to this crisis as they are the first frontline to receive and manage COVID-19 patients. In this multicenter multinational survey, we aim to assess the level of awareness and preparedness of hospital staff regarding COVID-19 all over the world.

**Methods:** From February to March 2020, the web-based or paper-based survey to gather information about the hospital staff's awareness and preparedness in the participants' countries will be carried out using a structured questionnaire based on the United States Centers for Disease Control and Prevention (CDC) checklist and delivered to participants by the local collaborators for each hospital. As of March 2020, we recruited 374 hospitals from 58 countries that could adhere to this protocol as approved by their Institutional Review Boards (IRB) or Ethics Committees (EC).

**Discussion:** The awareness and preparedness of HCWs against COVID-19 are of utmost importance not only to protect themselves from infection, but also to control the virus transmission in healthcare facilities and to manage the disease, especially in the context of manpower lacking and hospital overload during the pandemic. The results of this survey can be used to inform hospitals about the awareness and preparedness of their health staff regarding COVID-19, so appropriate policies and practice guidelines can be implemented to improve their capabilities of facing this crisis and other future pandemic-prone diseases.

## Introduction

The recent outbreak of Coronavirus disease (COVID-19) caused by a novel coronavirus (named SARS-CoV-2) has gained attention globally and has been recognized as a serious public health threat by the Centers for Disease Control and Prevention (CDC). The first case was detected in Wuhan City, Hubei Province, China and since then, the disease has spread rapidly (1). As of February 28, 2020, the World Health Organization (WHO) declared that the outbreak of COVID-19 as a Public Health Emergency of International Concern (PHEIC) with 62 countries now reporting 85,176 confirmed cases (79,250 of which have been in mainland China) and 2,919 deaths to date (2).

The SARS-CoV-2 is a novel strain of coronavirus emerging in the human population in the past two decades, preceded by the SARS-CoV outbreak in 2002 and the MERS-CoV outbreak in 2012 (3). The exact origin of this novel coronavirus and its precise disease mechanism has not been fully understood. At present, no antiviral medication or vaccine is approved for SARS-CoV-2 infection and the infected patients are managed with supportive care (1). The highly contagious capacity of SARS-CoV-2 led to rapid growth in the number of COVID-19 patients (4). As a result, hospital overload occurred in several regions where the SARS-CoV-2 infection became widespread in the community (5, 6). HCWs are at the core of the combat against COVID-19. Consequently, HCWs in most settings are overworked and more vulnerable to be infected with COVID-19. In Italy, by 22 March, 4,824 healthcare workers (HCWs) had been infected (9% of total cases) and 24 had died - these figures are worse than those observed in China (3,300 infected cases and 23 deaths among HCWs) (7). The awareness and preparedness of HCWs in response to the COVID-19 outbreak are of great importance not only to prevent disease contraction from the infected patients but also to help them cope with emergency situations and prevent further transmission.

To control the virus transmission in the healthcare facilities and protect the medical staff, the CDC in the United States and the WHO have developed the preparedness and prevention checklists of SARS-CoV-2 infection to be used by healthcare professionals (8, 9). However, the awareness and preparedness of medical staff against COVID-19 outbreaks around the world have not been reported.

## Methods and Analysis

- **Objectives:** This is a multicenter multinational survey aiming to assess the level of awareness of hospital staff regarding COVID-19 all over the world. It will also measure the level of preparedness of hospital staff in response to the crisis of COVID-19 and how will they react to limit and prevent further transmission.
- **Study design:** Cross-sectional study.
- **Time period:** February to March 2020.
- **Study Settings:** Any hospital in the world that can adhere to this protocol to conduct the survey as approved by its Institutional Review Board (IRB) or Ethics Committee (EC). Each hospital will have local collaborators.
- **Study population:** Healthcare providers in the hospitals including physicians, nurses, pharmacists, and others. We will enroll staff members who are or will be handling suspected cases in settings such as Emergency Department, Intensive Care Unit, Outpatient Department, Infectious Disease Clinic, Respiratory Disease Clinic, or any department designed to treat COVID-19 patients. We will exclude participants who cannot communicate in the vernacular of the translated questionnaire. We will also exclude staff who are on leave on the day of the survey.
- **Sample size calculation:** The survey will be conducted in a convenient selection of global hospitals. There will be no restriction on the number of hospitals per country or the number of participants per hospital.
- **Study instrument and questionnaire design process:** The survey will be carried out using a structured questionnaire adapted from the United States' CDC checklist (8). The original questionnaire will be developed in English, consisting of 2 sections with 32 questions in total. The first section covers 6 questions about demographic and personal medical aspects. The second section includes 26 questions assessing the awareness and preparedness of hospital staff regarding COVID-19. There are different types of questions in the questionnaire including yes/no questions, open-ended questions, and multiple-choice questions (MCQ).
- **Validation of questionnaire:** The original questionnaire will be carefully revised by a panel of healthcare professionals that includes one WHO consultant, three epidemiologists, five physicians; three members are native English speakers. A pilot survey will be conducted by 30 international HCWs to ensure the validity of the questionnaire. This validation aims to evaluate the time needed to complete the questionnaire and assure that all the questions and sections of the questionnaire are phrased clearly and appropriately for comprehension and to avoid bias that might otherwise. After the pilot survey, the original questionnaire will be modified if needed. The local team members in each participated country are responsible for its translation into their native languages. For the translated questionnaires, forward and reverse translation will be performed to ensure their accuracy. A pre-test of the questionnaire by 5 native speakers will also be conducted for the translated version. The questionnaires will be then modified if required.
- **Survey conduct:** To gather information about the hospital staff's awareness and preparedness in the participants' countries, we will develop an online questionnaire using SurveyMonkey© that limits one-time participation per unique IP address. However, participants can choose to use hard copies prepared by the local collaborators for each hospital.
- **Coordination and participating sites of the survey:** Our global research team will include several medical students and doctors from many countries around the world. We will use social networks and send recruitment emails for inviting the collaborators and coordinators around the world to participate in the study.
  **Local Site Collaborators:** Two or three collaborators are required for each local site hospital. Local collaborators will be specifically responsible for:
  1. Obtaining local audit, special exemption, or research approval (IRB/EC approval).
  2. Listing all departments that are or will handle the patients (Emergency Department Intensive Care Unit, Outpatient Department, Infectious Disease Clinic, Respiratory Disease Clinic, or any department designed to treat COVID-19 patients).
  3. Reporting the number of doctors, nurses, other HCWs of each department. If there are only a few staff in a particular department, the collaborators will assign that department as “others.”
  4. Preparing the hard print of the survey questionnaire provided by our coordinator.
  5. Distributing the questionnaire to the head of the department and collect it within 1 day, report the number of doctors, nurses, other workers of each department that is available on that day.
  6. Scanning all collected questionnaires and sending a zip file to the corresponding coordinator via email using Google folder or via email.
  7. Keeping all the hard copies of the collected questionnaires for at least 5 years and protecting the information inside those copies.
  The survey questionnaire is anonymous and participant identification numbers will be used rather than any personal identifiers. The site collaborators are fully responsible for the accuracy and any misconduct of research. Data cannot be published without prior written permission from Dr. Nguyen Tien Huy. Local collaborators may request permission to publish in a local journal after the main publication.
  **Project coordinators:** Each coordinator is responsible for 3–5 hospital sites and:
  1. Recruiting 3–5 local site hospitals.
  2. Supporting translation of the questionnaire (both forward and reverse translations) to the local language and conduct a pre-test with the questionnaire.
  3. Assisting and communicate between the project management team and local collaborators.
  4. Checking evidence of action and quality of the data scanning provided by the collaborators.
  **Project managing team:**
  1. Writing the protocol and developing the questionnaire.
  2. Recruiting coordinators and follow all of their steps.
  3. Importing data in an online forum and collect them in spreadsheets to prepare them for the coding process.
  4. Analyzing data and writing a report.
- **Data management:** The collected data will be organized by Google Sheets and collected in an Excel spreadsheet. The survey will be completely anonymous. Hard copies of questionnaires will be scanned and uploaded to a Google drive encrypted by a password. Only the management team will be able to access all data. Data entered Google Sheets will be quality-checked by a researcher to ensure accuracy.
- **Data analysis:** Data collected will be exported to the Microsoft Excel sheet. Every respondent will be given an overall score for awareness and preparedness. The awareness of HCWs will be assessed using MCQ questions of 4 topics regarding COVID-19 including symptoms, diagnosis, mode of transmission, preventive measures. A score of 10 will be given for each topic. The preparedness of HCWs will be evaluated using yes/no questions, a score of “1” will be given for the option “yes,” and a score of “0” will be given for the option “no” or “I don't know.”
  Descriptive statistics will be performed and variations among international healthcare settings will be assessed by categorizing countries with participating hospitals into lower-income, upper and lower middle-income, and higher-income groups, according to the World Bank's classification of Gross national income (GNI) per capita (10). A hierarchical logistic regression multivariate analysis will be applied to adjust the influence of GNI on the awareness and preparedness scores for confounding variables. Model coefficients will be presented as odds ratio (OR) and 95% confidence intervals. All analyses will be performed using the R Foundation Statistical Program version 3.6.3.
- **Timetable**

**Table:** 

**Time**	**List of activities**
31/01/2020 - 02/02/2020	• Establish the research team • Develop the survey questionnaire • Develop the survey protocol
03/02/2020 - 15/02/2020	• Revise the protocol, questionnaire (pre-test and post-test) • IRB approval by Nagasaki University • Recruit coordinators and collaborator
06/02/2020 – 03/03/2020	• Translate questionnaire to local language • IRB approval by local hospitals
16/02/2020– 16/03/2020	• Conduct the survey, import data
16/03/2020– 16/04/2020	• Data analysis and report
- **Financial support:** Self-supported at each site.
- **Authorship:** Each author needs to fulfill the criteria listed in this protocol, qualify as a co-author in the publication. The task must be finished before the deadline shown in the Timetable (Item L). All authors will be listed as a group of collaborators as described in previous work (Figure 1) (11). In addition, the author's contributions will be also recorded as presented in Figure 2 of the previous publication (11). All data cannot be published without permission from Dr. Nguyen Tien Huy. Local collaborators may request permission to publish in a local journal after the main publication.

**Figure 1:**
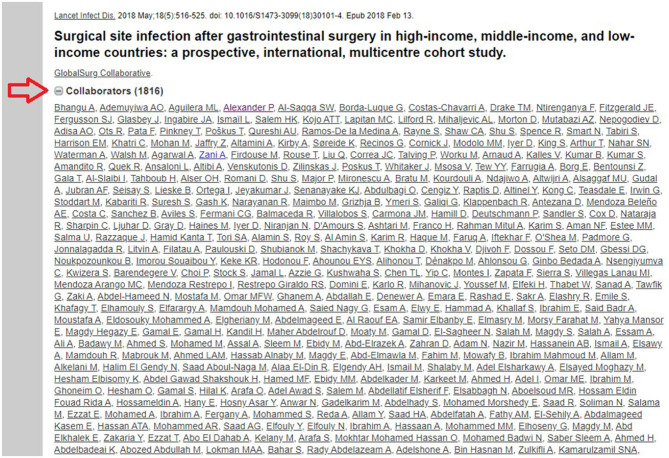
Illustration for the method of listing collaborators in a global multicentre study (11).

**Figure 2:**
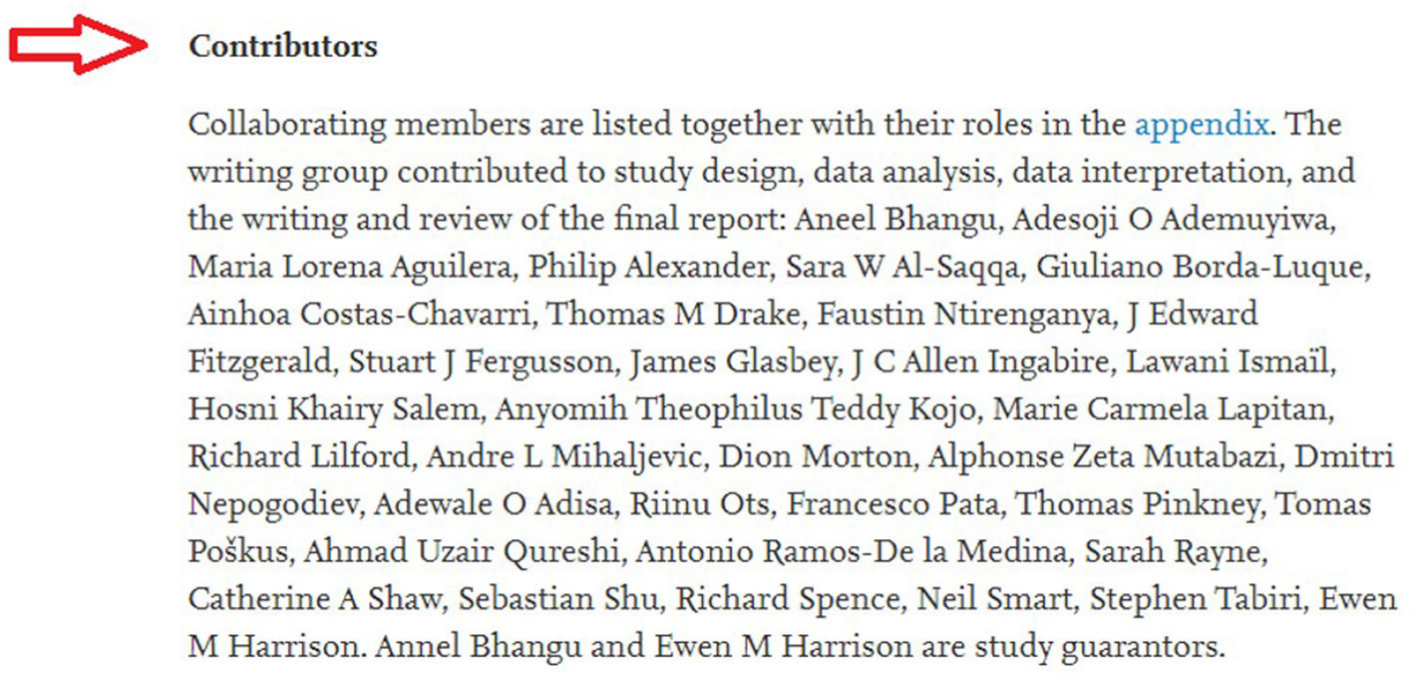
Illustration for the method of listing authors' contribution in a global multicentre study (11).

## Discussion

The awareness and preparedness of HCWs against an outbreak are crucial to public health and their issues have been raised universally. COVID-19 outbreak reached a very high transmission rate worldwide and the evaluation of front-liners dealing with such an outbreak is important. The awareness and preparedness level of HCWs play an important role in the control of a public health crisis (12). This protocol provides a way to conduct a global multicenter study regarding the level of awareness and preparedness of global HCWs in combating the crisis of COVID-19 pandemic through collaboration with participants from many hospitals around the world and can recruit medical staff to participate in the survey within a short-term framework, giving results of a global multi-center survey in a short time. As a result, the study can quickly provide a picture of global HCWs' awareness and preparedness for the spread and outbreak of the COVID-19 pandemic.

The research can cover a large number of countries in different regions, thus the overall survey provided important and useful information about the preparedness of hospitals and awareness of the staff against the country.

This survey will provide a final awareness and preparedness score that will reflect the hospital's state in regard to dealing with the COVID-19 pandemic. This score will help hospitals as a consequence to consider implementing policies and practice guidelines in case their facilities deemed to be unprepared, and will also give them information about their staff during the pandemic which will reflect their capabilities of facing other future pandemic-prone diseases.

This study was mainly conducted online among HCWs during a time when an alarming number of COVID-19 cases were being reported globally, and this might limit generalization. Also, the survey was conducted in the first few months of the pandemic where not enough information about the virus transmission and pathogenicity were available which might have an effect on participants' answers. Despite these limitations, we believe that our study is unique and the first to provide information about the awareness and preparedness of numerous HCWs during the COVID-19 pandemic.

## Ethics and Dissemination

### Ethics Approval and Consent to Participate

This project protocol was approved by the Ethics Committee of Graduate School of Tropical Medicine and Global Health, Nagasaki University, Japan (NU_TMGH_2020-111-0).

**Plan for getting informed consent and protecting confidentiality:** All the respondents of the survey will fill a written informed consent embedded on the first page of the questionnaire. If the participant answers “YES” to the first question of the form, he/she automatically agrees to participate and will begin the survey. By using the skip-logic survey method, users who disagree with the informed consent question will be directed to the end of the survey. No respondent is forced to participate in the survey and their participation is based on their agreement that can be withdrawn at any time.
**Autonomy:** All participants have the right to leave a specific question unanswered or withdraw from the survey any time if they feel uncomfortable answering any question. In addition, no one even the research team will know individual answers to this questionnaire.
**Risks and benefits for the participants:** Data collected from this survey will play an important role in future reactions to fatal virus outbreaks. It will be used by a variety of researchers from different countries to improve the preparedness of different hospitals for outbreaks. This will play a crucial role in the early management and prevention of viral outbreaks in other areas. It will also play an important role in decreasing the response time to emergency cases at the hospital. We confirm that there are no risks associated with participating in this survey. As our study does not report individual results for each hospital, there will be no risk associated with the hospital's responsibility for their HCWs' awareness and preparedness regarding COVID-19 from our study results. Any unexpected risks that may occur during the survey will be immediately explained to both participants and the ethical committee. The responses collected from this survey are confidential and will not be revealed under any condition. In addition, the survey will be completely anonymous regarding participants and hospital names. Responses collected from this will be reported as collective combined data.

## Ethics Statement

The studies involving human participants were reviewed and approved by the Ethics Committee of Graduate School of Tropical Medicine and Global Health, Nagasaki University, Japan. The patients/participants provided their written informed consent to participate in this study.

## Author Contributions

NH raised the idea and took responsibility for the work integrity. AQ, SN, and AG designed the research study and drafted the protocol. ML, HS, SA, and NV developed and validated the questionnaire. CS, PL, RC, SC, and KH revised the questionnaire and the protocol. AS wrote the invitation letter with informed consent to recruit the coordinators and collaborators. TA-A, AS, VH, GT, MH, SD, MM, SA, NN, and SI recruited and supervised the local coordinators to collect data. SG, NV, and AS will analyze the data. All authors contributed to the article and approved the submitted version.

## Conflict of Interest

The authors declare that the research was conducted in the absence of any commercial or financial relationships that could be construed as a potential conflict of interest.

## Abbreviations

CDC: centers for disease control and prevention
COVID-19: Coronavirus Disease 2019
EC: ethics committee
HCWs: healthcare workers
HDI: human development index
IRB: institutional review board
MCQ: multiple-choice question
PHEIC: public health emergency of international concern
WHO: world health organization.
